# Challenges and successful management of subglottic tracheal stenosis in a 2‑year‑old child: A case report and a mini‑review of the literature

**DOI:** 10.3892/mi.2023.113

**Published:** 2023-09-20

**Authors:** Fahmi H. Kakamad, Mariwan L. Fatah, Rezheen J. Rashid, Karzan M. Hasan, Bilal A. Mohammed, Honar Othman Kareem, Sarwat T. San Ahmed, Khdir Hussein Hamad Khoshnaw, Sanaa O. Karim, Berun A. Abdalla, Sarhang Sedeeq Abdullah

**Affiliations:** 1 Department of Scientific Affairs, Smart Health Tower, Sulaimani, Kurdistan 46001, Iraq; 2 College of Medicine, University of Sulaimani, Sulaimani, Kurdistan 46001, Iraq; 3 Kscien Organization for Scientific Research, Sulaimani, Kurdistan 46001, Iraq; 4 Department of Oncology, Hiwa Hospital, Sulaimani, Kurdistan 46001, Iraq; 5 Sulaimani Teaching Hospital, Sulaimani, Kurdistan 46001, Iraq; 6 Pediatric Department, Ranya Maternity and Pediatric Teaching Hospital, Sulaimani, Kurdistan 46001, Iraq; 7 College of Nursing, University of Sulaimani, Sulaimani, Kurdistan 46001, Iraq

**Keywords:** tracheal stenosis, bronchoscopy, tracheostomy

## Abstract

Tracheal stenosis is a narrowing of the windpipe that can lead to shortness of breath, stridor and even suffocation. The present study reports the clinical course of a patient with this condition in an aim to help clinicians obtain more information about this rare condition and identify potential treatment options. A 2-year-old female child presented with progressive shortness of breath and stridor. She was initially managed with tracheostomy; however, this was unsuccessful in relieving the stenosis. Subsequent interventions, including rigid bronchoscopy and dilatation were successful in relieving the condition. A benign hypertrophy of the bronchial wall was identified through biopsy. The patient was treated with steroids and antibiotics, and she experienced a marked improvement in symptoms and remained asymptomatic after a 1-year follow-up. Tracheal stenosis is a rare, yet serious condition that may be life-threatening. Thus, the early diagnosis and treatment of this condition are essential in order to improve outcomes.

## Introduction

Tracheal stenosis is a rare condition that involves the narrowing of the tracheal lumen, causing significant respiratory distress and potentially life-threatening complications (1). It can occur due to a variety of factors, such as congenital abnormalities, acquired conditions, or traumatic injuries with a reported incidence of 1 in 6,400 live births for congenital type, with a mortality rate as high as 79% before the advent of current surgical techniques (2). While tracheal stenosis is more commonly observed in adults, its occurrence in pediatric populations is less frequent. The diagnosis and treatment of tracheal stenosis in children pose unique challenges that require a comprehensive understanding of the condition and its management strategies (3). The present study describes the clinical course of a 2-year-old female child diagnosed with tracheal stenosis. The present study aimed to provide valuable insight into the diagnostic and therapeutic complexities encountered during the treatment of this condition.

## Case report

### Patient information

A 2-year-old female child, who had previously undergone a tracheostomy and polypectomy at another medical center, was referred to the Smart Health Tower, Sulaimani, Iraq due to progressively worsening stridor and shortness of breath. The symptoms gradually increased in intensity, eventually reaching a severe level, where the child experienced episodes of suffocation.

### Clinical findings

Due to the urgency of the case, no physical examination had been conducted at the previous center. An emergency tracheostomy was thus immediately performed, which provided temporary relief. Later, due to the persistent nature of the condition, the case was referred to the Cardiothoracic Department at Smart Health Tower (Sulaimani, Iraq).

### Diagnostic assessment

**Figure 1:**
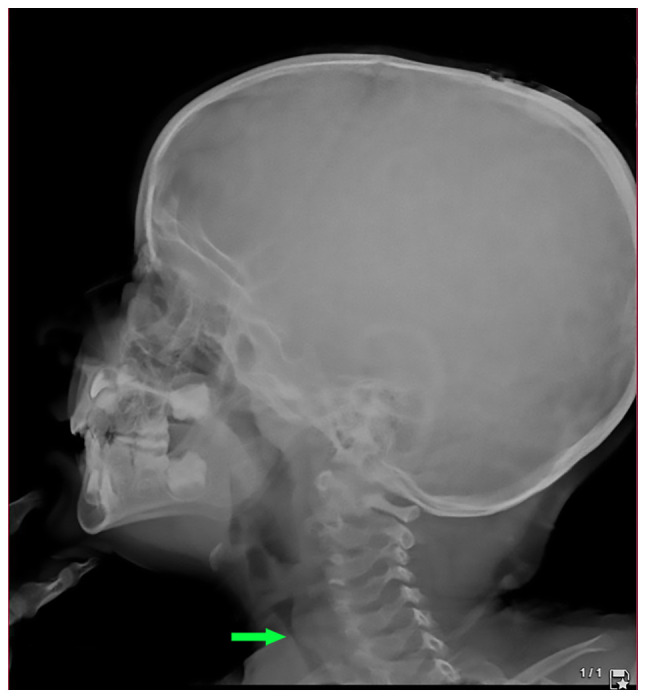
A pre-tracheostomy lateral post-nasal space radiograph illustrating a bulging from a posterior cervical tracheal wall (arrow) at the level of C5, resulting in severe narrowing of the tracheal air column.

**Figure 2:**
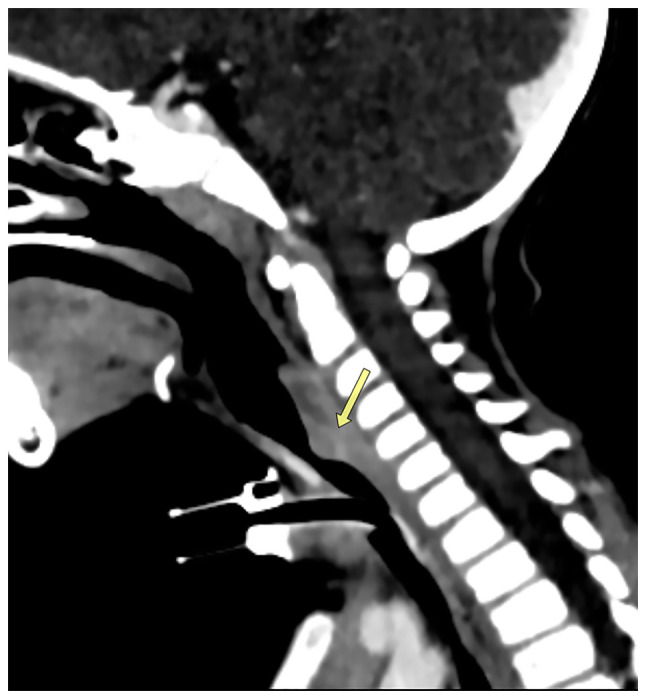
A sagittal contrast-enhanced post-tracheostomy neck computed tomography scan illustrating severe luminal stenosis of the cervical trachea (~70%) by enhancing a mass that protrudes from the posterior tracheal wall (arrow).

A lateral post-nasal space X-ray was performed, which illustrated tracheal stenosis (Fig. 1). A computed tomography (CT) scan was conducted and confirmed severe subglottic stenosis and a narrowing of the tracheal region just below the vocal cords (Fig. 2).

### Therapeutic intervention

The patient had previously undergone a rigid laryngoscopy to remove a vocal cord polyp at another medical center. However, the intervention did not yield the desired clinical benefit, and the child's symptoms persisted. Under general anesthesia, a rigid bronchoscopy procedure was performed. The bronchoscope was inserted through the mouth and into the trachea. The narrowed section of the trachea, proximal to the stenosis, was identified and forcefully dilated using a combination of scopes and dilators of various sizes. A biopsy sample was collected from the hypertrophied bronchial wall, and the pathological analysis confirmed benign hypertrophic tissue. Following 1 week of tracheostomy weaning, during which the child gradually transitioned to breathing independently without the aid of the tracheostomy tube, a new symptom emerged. The child developed stridor, particularly noticeable during sleep. Concerned about the recurrence of respiratory distress, a subsequent rigid bronchoscopy was performed, and the trachea appeared normal. However, some features suggestive of tracheomalacia were identified. The condition was confirmed to be congenital due to the presence of tracheomalacia. Following decannulation, the patient was hospitalized for 3 days to monitor for any emerging dyspnea. An echocardiography was performed to rule out any associated cardiovascular abnormalities, and this revealed no significant findings (data not shown). The child was prescribed a treatment regimen consisting of steroids and antibiotics. Notably, the patient responded favorably to the medical intervention, with a significant improvement in symptoms.

### Follow-up

During a 1-year follow-up period, the child remained asymptomatic, indicating successful management of tracheal stenosis caused by tracheomalacia.

## Discussion

Tracheal stenosis, medical condition whose incidence among infants is rare, is characterized by the narrowing of the trachea, leading to respiratory distress and potential complications (4). It is crucial for medical professionals to have a thorough understanding of this condition and its associated complications in order to ensure appropriate treatment and care for affected infants.

Common symptoms of tracheal stenosis include shortness of breath and stridor, a high-pitched sound during breathing. In severe cases, respiratory failure and asphyxiation can occur, underscoring the need for early diagnosis and appropriate management (4).

The incidence of tracheal stenosis is estimated to be 1 in 6,400 live births, emphasizing its rarity and the need for heightened awareness (2). To diagnose tracheal stenosis, a comprehensive evaluation is required, incorporating imaging techniques such as CT scans and bronchoscopies, alongside a meticulous medical history and physical examination (5). The key differential diagnosis is foreign body aspiration (6).

The treatment of tracheal stenosis is dependent on the severity and underlying cause of the condition. Several treatment options are available to alleviate symptoms and improve airway function:

Airway dilation involves the insertion of a balloon through a bronchoscope into the narrowed area of the trachea. The balloon is then inflated to expand the airway and enhance airflow (7). Balloon dilation has been proven to be a secure and efficient palliative technique for addressing both congenital and acquired tracheal and bronchial stenosis. Notable enhancements in symptoms and the widening of the airway can be observed, although these improvements may only be temporary. In order to achieve a lasting remedy, a series of sequential dilation procedures may be required (7).

In certain cases, a stent may be positioned within the trachea to maintain its patency. This can be achieved using a bronchoscope or through an open surgical procedure (8). According to previous research, >90% of patients achieve the rapid alleviation of dyspnea rather than the complete resolution of tracheal stenosis. To assess the efficacy of silicone stent placement, various factors are considered, including the Borg scale, lung function, CT measurements of stenosis, curative rates (successful stent removal) and stability rates (8).

Laser therapy is utilized to remove excess or scar tissue causing tracheal narrowing. It is typically performed with the assistance of a bronchoscope (9). Neodymium:Yttrium-Aluminium-Garnet (Nd:YAG) laser photocoagulation has demonstrated effectiveness in managing tracheal stenosis caused by benign lesions, with the exception of subglottic region involvement. The procedure is associated with minimal morbidity and no mortality risks (10). Severe cases of tracheal stenosis may necessitate surgical intervention to remove the narrowed portion of the trachea and reconstruct the airway. Various techniques, such as tracheal resection and anastomosis or tracheal reconstruction with grafts, may be employed (11). The most effective treatment for benign tracheal stenosis is surgery, specifically tracheal resection, and end-to-end anastomosis. A previous study demonstrated a success rate of 88% for surgery in addressing airway stenosis, with an almost zero complication rate (12). However, only a small percentage of patients (<10%) with tracheal stenosis are suitable candidates for surgery and resection due to the significant risks associated with anesthesia and surgery, as well as the length of the tracheal resection and the extent of the lesion (13). In the case in the present study, the condition was initially suspected to be caused by a polyp on the vocal cord; thus, the patient, at another center, underwent an emergency tracheostomy and polypectomy. The condition was temporarily relieved but later emerged again; thus, the case was then referred to the authors. After conducting a bronchoscopy, the case was diagnosed as tracheomalacia. Proper workups such as echocardiography were subsequently performed to exclude any associated cardiovascular abnormalities.

In certain instances, medications such as corticosteroids may be prescribed to mitigate inflammation and alleviate symptoms. However, medical management alone is generally not a long-term solution and is often used in conjunction with other treatments (14). By implementing these treatment options, medical professionals aim to relieve symptoms, restore proper airway function and enhance the quality of life of individuals with tracheal stenosis. The case described herein was prescribed a treatment regimen consisting of steroids and antibiotics, as mitomycin was not available at that time, and the patient responded favorably to the medical intervention. The limitation of the present study was that the authors were not able to retrieve any data or information regarding how the tracheostomy procedure was conducted or which protective method was applied, as it was performed at another center. In the present study, all the references have been filtered to avoid citing non-peer-reviewed data (15).

In conclusion, tracheal stenosis is a rare condition that poses significant challenges to affected infants. The diagnosis involves a comprehensive evaluation, considering imaging techniques and careful assessment of medical history. Various treatment modalities, including airway dilation, stent placement, laser therapy, surgery and medical management may be employed to alleviate symptoms and improve outcomes. Continued research and collaboration among healthcare professionals are essential to further enhance our understanding of this condition and optimize treatment strategies.

## Data Availability

The datasets used and/or analyzed during the current study are available from the corresponding author on reasonable request.
